# Abnormal activation of MAPKs pathways and inhibition of autophagy in a group of patients with Zellweger spectrum disorders and X-linked adrenoleukodystrophy

**DOI:** 10.1186/s13023-023-02940-x

**Published:** 2023-11-16

**Authors:** Vincenza Gragnaniello, Daniela Gueraldi, Andrea Puma, Anna Commone, Chiara Cazzorla, Christian Loro, Elena Porcù, Maria Stornaiuolo, Paolo Miglioranza, Leonardo Salviati, Ronald J. A. Wanders, Alberto Burlina

**Affiliations:** 1 Division of Inherited Metabolic Diseases, Department of Women’s and Children’s Health, University - Hospital of Padova, Padua, Italy; 2 Division of Inherited Metabolic Diseases, Department of Women’s and Children’s Health, University of Padua, Padua, Italy; 3 Clinical Genetics Unit, Department of Women’s and Children’s Health, and Myology Center, University of Padova, Padua, Italy; 4 Laboratory Genetic Metabolic Diseases, Department of Clinical Chemistry, Amsterdam University Medical Center, University of Amsterdam, Amsterdam, The Netherlands; 5 Department of Pediatrics, Emma Children’s Hospital, Amsterdam University Medical Center, University of Amsterdam, Amsterdam, The Netherlands

**Keywords:** Peroxisomal disorders, Zellweger spectrum disorders, X-linked adrenoleukodystrophy, Oxidative stress, Autophagy, Mitogen-activated protein kinases (MAPKs), Antioxidants, LC3, p38, JNK

## Abstract

**Background:**

Zellweger spectrum disorders (ZSD) and X-linked adrenoleukodystrophy (X-ALD) are inherited metabolic diseases characterized by dysfunction of peroxisomes, that are essential for lipid metabolism and redox balance. Oxidative stress has been reported to have a significant role in the pathogenesis of neurodegenerative diseases such as peroxisomal disorders, but little is known on the intracellular activation of Mitogen-activated protein kinases (MAPKs). Strictly related to oxidative stress, a correct autophagic machinery is essential to eliminated oxidized proteins and damaged organelles. The aims of the current study are to investigate a possible implication of MAPK pathways and autophagy impairment as markers and putative therapeutic targets in X-ALD and ZSDs.

**Methods:**

Three patients with ZSD (2 M, 1 F; age range 8–17 years) and five patients with X-ALD (5 M; age range 5- 22 years) were enrolled. A control group included 6 healthy volunteers. To evaluate MAPKs pathway, p-p38 and p-JNK were assessed by western blot analysis on peripheral blood mononuclear cells. LC3II/LC3I ratio was evaluated ad marker of autophagy.

**Results:**

X-ALD and ZSD patients showed elevated p-p38 values on average 2- fold (range 1.21- 2.84) and 3.30-fold (range 1.56- 4.26) higher when compared with controls, respectively. p-JNK expression was on average 12-fold (range 2.20–19.92) and 2.90-fold (range 1.43–4.24) higher in ZSD and X-ALD patients than in controls. All patients had altered autophagic flux as concluded from the reduced LC3II/I ratio.

**Conclusions:**

In our study X-ALD and ZSD patients present an overactivation of MAPK pathways and an inhibition of autophagy. Considering the absence of successful therapies and the growing interest towards new therapies with antioxidants and autophagy inducers, the identification and validation of biomarkers to monitor optimal dosing and biological efficacy of the treatments is of prime interest.

## Introduction

Peroxisomes are ubiquitous intracellular organelles with an essential role in multiple metabolic pathways. They are responsible for saturated very long chain fatty acids (VLCFAs, > C22:0) and branched chain fatty acids (phytanic and pristanic acids) oxidation, degradation of pipecolic acid as well as for the synthesis of bile salts, polyunsaturated fatty acids and plasmalogens [1]. Moreover, they are implicated in hydrogen peroxide (H_2_O_2_) detoxification by the enzymatic antioxidant catalase [2].

Peroxisomal disorders are a group of inherited metabolic diseases caused by either a defect in peroxisome biogenesis or a deficiency of one or more peroxisomal enzyme activities [3]. Zellweger Spectrum Disorders (ZSD) include a group of autosomal recessive peroxisome biogenesis disorders (PBD), caused by bi-allelic mutations in different PEX genes which code for a diverse group of proteins called peroxins required for the proper biosynthesis of peroxisomes [4]. The overall incidence is about 1 in 50,000 to 100,000 live births [5]. The main clinical features include dysmorphic features, visual and hearing impairment, cerebral malformations, white matter disorders, hepatomegaly and renal cysts [6, 7].

X-linked adrenoleukodystrophy (X-ALD) represents the most frequent peroxisomal disorder, with an incidence of 1:17,000–1:20,000 [8]. It is due to mutations in the ABCD1 gene (Xq28), coding for a transmembrane protein that imports VLCFAs across the peroxisomal membrane for its degradation [9]. The disorder primarily affects the adrenal cortex and the nervous system. Three core clinical syndromes are usually discriminated which include: the severely progressive form dominated by leukodystrophy (cerebral X-ALD, CALD), the progressive form with myeloneuropathy as major abnormality affecting patients in adulthood (adrenomyeloneuropathy, AMN) and primary adrenocortical insufficiency. Myeloneuropathy is the most common phenotype in affected females [10].

Patients affected by X-ALD and ZSD show increased plasma VLCFAs (mainly C26:0, C24:0/C22:0 ratio and C26:0/C22:0 ratio) and C26:0-Lysophosphatidylcholine (C26:0-lysoPC) [11]. The diagnosis is confirmed by genetic testing. At present, treatment focuses largely on symptomatic or supportive therapies [12]. For cerebral forms of X-ALD, allogeneic hematopoietic stem cell transplantation (HSCT) can be effective in childhood CALD if performed in neurologically asymptomatic boys with early brain magnetic resonance imaging (MRI) lesions [13–15].

In both ZSD and X-ALD, the central nervous system (CNS) is one of the main tissues affected and the lack of effective and curative treatment may probably reflect the fact that the pathophysiology of the brain injury in these disorders is poorly known.

The CNS is highly susceptible to oxidative damage due to the relatively low activity of antioxidant defenses, high iron content, high lipid content and high oxygen consumption [16]. Oxidative stress and inflammation may also have a significative role in the pathogenesis of peroxisomal disorders. Peroxisomes do produce intracellular H_2_O_2_ by virtue of the fact that peroxisomes contain multiple oxidases which turn molecular oxygen into H_2_O_2_ and their metabolism is highly connected to mitochondrial metabolism in the regulation of cellular redox homeostasis [17, 18]. Furthermore, the excess of VLCFAs are incorporated into complex lipids of cell membranes, which may contribute to the activation of inflammatory responses and oxidative stress [6, 19]. Mitochondrial dysfunction and excessive reactive oxygen species (ROS) production have been described in both X-ALD and ZSD [20–22].

Because of the very short half-life and rapid reactivity of ROS, there is currently no reliable method or technology to directly measure its level. Consequently, redox levels are mostly indirectly reflected by measuring antioxidant capacity (e.g. superoxide dismutase, catalase, glutathione peroxidase), or markers of oxidative damage to DNA, proteins and lipids [23].

To our knowledge, there are no studies on intracellular pathways activated by ROS, specifically on the activation of Mitogen-activated protein kinases (MAPKs). For this reason, we decided to investigate the possible implication of MAPK pathways as markers and putative therapeutic targets in X-ALD and ZSD. Given the close link between oxidative stress/MAPKs and autophagy, we also investigated autophagy impairment, previously described in X-ALD but understudied in ZSD.

## Material and methods

### Study population

Three patients with ZSD and five patients with X-ALD were enrolled. All caregivers gave informed consent to participate in the study which was conducted in accordance with the principles of the Declaration of Helsinki. The biochemical diagnosis was established by increased levels of plasma VLCFAs, measured by gas chromatography, and confirmed by molecular examination. C26:0-lysoPC was measured by LC- MS/MS on dried blood spot (Tables 1 and 2).

**Table 1:** Patients’ data at diagnosis

Patient	Age at diagnosis	Disease	Variants (DNA)	Variants (protein)	Gene	C26:0 μmol/L (nv 0.25–0.65)	C26:0/C22:0 (nv 0.01–0.03)
1	2 months	ZSD	c.2097dupT c.2528G > A	p.Ile700Tyrfs*42 p.Gly843Asp	PEX1	7.67	0.31
2	1 month	ZSD	c.2097dupT c.2528G > A	p.Ile700Tyrfs*42 p.Gly843Asp	PEX1	3.76	0.13
3	5 years	ZSD	c.292C > T c.419_427del	p.Arg98Trp p.Val140_Gly142del	PEX26	2.67	0.08
4	9 years	X-ALD	c.526_528del	p.[Ser176del]	ABCD1	4.3	0.10
5	3 years	X-ALD	c.526_528del	p.[Ser176del]	ABCD1	5.33	0.18
6	14 years	X-ALD	c.1679C > T	p.[Pro560Leu]	ABCD1	3.29	0.08
7	10 years	X-ALD	c.1679C > T	p.[Pro560Leu]	ABCD1	2.4	0.06
8	3 years	X-ALD	c.[652C > T; 664G > T]	p.[Pro218Ser; Val222Leu]	ABCD1	1.17	0.10

**Table 2:** Patients’ biochemical data at the moment of the study

Patient	Age (years)	C26:0 μmol/L (nv 0.25–0.65)	C26:0/C22:0 (nv 0.01–0.03)	C26:0-LysoPC μmol/L (nv < 0.08)
1	17	2.54	0.10	0.52
2	17	4.05	0.13	0.98
3	8	4.06	0.14	1.03
4	12	2.74	0.04	0.51
5	5	2.42	0.07	0.7
6	22	1.43	0.03	0.38
7	18	0.93	0.03	0.26
8	14	0.87	0.02	0.15

NFS Neurologic function score (NFS), a 25-point scale used to evaluate the severity of neurologic dysfunction. Presymptomatic are scored = 0. IQ intelligence quotient measured by Wechsler Intelligence Scales. MRI Magnetic resonance imaging, Loes score severity system is a 34-point based severity scale for brain MRI that takes into account the location and extent of disease and the presence of focal and/or global atrophy (Loes et al. 2004). GTE/GTO glycerol trioleate/glycerol trierucate

The 3 patients with ZSDs, 1 female and 2 males, were aged between 8 and 17 years (Table 3). All had intellectual disability, hearing loss, and visual loss. Patient 2 additionally had epilepsy, patients 1 and 3 hepatomegaly. All patients were supplemented with vitamin D and calcium. Patient P2 was treated with antiepileptics (carbamazepine and clobazam) and had been supplementing with vitamin E (200 mg/day), coenzyme Q 10 (200 mg/day) and docosahexanoic acid (DHA) (800 mg/day) since the age of 6.5 years. Patients with adrenoleukodystrophy, all males, ranged in age from 5 to 22 years. Patient’s characteristics are summarized in Table 4. Patients P4 and P5 and patients P6 and P7 were two pairs of brothers. All but one patient (P8) had Addison's disease and were on replacement therapy with hydrocortisone.

**Table 3:** ZSDs patients’ characteristic

Patient	Age (years)	Clinical features	C26:0 μmol/L (nv 0.25–0.65)	C26:0/C22:0 ratio (nv 0.01–0.03)	C26:0-LysoPC μmol/L (nv < 0.08)	Treatment
1	17	Neonatal hepatopathy, severe cognitive and motor disability, hearing and visual impairment	2.54	0.10	0.52	Calcium, Vitamin D, Oil mixture GTE/GTO
2	17	Neurodevelopmental delay, severe cognitive and motor disability, hearing and impairment, epilepsy	4.05	0.13	0.98	Carbamazepine, clobazam, bisphosphonate Vitamins (Vit E, D, Fe), CoQ, DHA
3	8	Neurodevelopmental delay, cognitive disability, hearing and visual impairment, hepatomegaly	4.06	0.14	1.03	NAC, calcium and vitamin D

**Table 4:** X-ALD patients’ characteristics

Patient	Age (years)	Phenotype	NFS	IQ	Behavioral and other symptoms	MRI (Loes score)	Treatment
4	12	Adrenal insufficiency	0	118	Hyperactivity	Normal (0)	Hydrocortisone, fludrocortisone Oil mixture GTE/GTO
5	5	Adrenal insufficiency	0	108	Hyperactivity	Normal (0)	Hydrocortisone Oil mixture GTE/GTO
6	22	Adrenal insufficiency, CALD	0	107	Impulsivity, adaptive and social difficulties	Corpus callosum genu and white matter of frontal lobes, internal capsule (6)	Oil mixture GTE/GTO Hydrocortisone
7	18	Adrenal insufficiency, CALD	0	97		Corpus callosum genu (1)	HSCT, hydrocortisone
8	14	CALD	3	55	Hyperactivity, visuospatial impairment and dyspraxia	Corpus callosum genu and splenium, optic tract, parieto-occipital and fronto-temporal white matter (11)	HSCT

Patients P6, P7 and P8 had CALD with abnormal brain MRI. Patients P4 and P5 had normal MRI at the time of the study, but P5 developed white matter abnormalities at cerebral MRI one year later. All presented normal cognitive performance, except P8, who had moderate cognitive disability (IQ 55).

At the time of sample collection, two patients with X-ALD (P7 and P8) had received hematopoietic stem cell transplantation (HSCT) for 1 month and 2 years, respectively.

All X-ALD patients were treated with a mixture of glycerol trioleate/glycerol trierucate (GTO/GTE) containing alfa-lipoic acid, L-glutathione and vitamin E.

Six healthy adult volunteers (26–32 years) were recruited to set up a control group.

### Sample collection

A blood sample was obtained by venous puncture with heparinized vials. Whole blood was centrifuged at 3000 rpm, plasma was used for routine biochemical analysis and measure of plasma VLCFAs. The cell pellet, which is usually discarded, was used to isolate peripheral blood mononuclear cells (PBMCs) by density gradient centrifugation on Ficoll (purchased from Cytiva). The pellet thus obtained was frozen at − 80 °C until analysis.

### Immunoblotting

PBMC extracts were resuspended in RIPA buffer (phosphate-buffered saline PBS with 1% NP-40, 0.5% sodium deoxycholate, 0.1% sodium dodecyl sulfate SDS) supplemented with 1% protease inhibitors (Thermo Fisher), disrupted by vortexing and centrifuged at 1300 rpm for 30 min at 4 °C. Protein concentration was measured by BCA (bicinchoninic acid) assay according to manufacturer’s instructions (PierceTM BCA Protein Assay kit, Thermo Fisher). Equal amounts (20 ug of protein) of extracts were heat-denatured for 5 min at 95 °C and subjected to sodium dodecyl sulfate polyacrylamide gel electrophoresis (4–15% polyacrylamide). PBMC extracts from healthy subjects were run in parallel for comparison. Proteins were transferred to a nitrocellulose membrane, that was incubated with blocking solution (Everyblot, Bio-Rad) for 5 min. Then, it was incubated with the primary antibody (anti-p-p38, anti-p-JNK, anti-LC3 rabbit polyclonal antibodies) overnight at 4 °C and subsequently with secondary antibodies (horseradish peroxidase HRP conjugated antirabbit IgG) for 1 h. Immunoreactive proteins were detected by chemiluminescence (ECL, Bio-Rad). The GAPDH peptide allowed comparison of different samples. All reagents (blocking solution, precast gels, nitrocellulose membranes, TBS-Tween 20, ECL) and instruments (Transblot, Chemidoc) were from Bio-Rad. Primary antibodies were from Cell Signaling (p-JNK, p-p38) or Biorad (LC3, GAPDH) and used with a dilution of 1:1000. Secondary antibodies were from Biorad and used with a dilution of 1:2000. Quantitative analysis of band intensity was performed using Image Lab.

## Results

### MAPK pathways

**Fig. 1:**
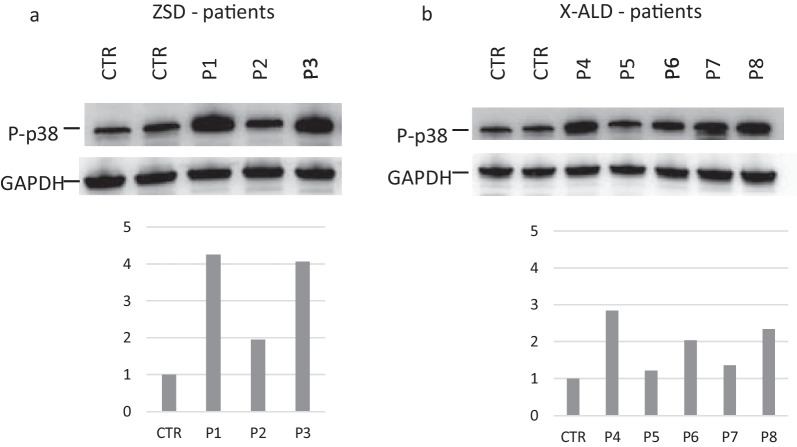
p-p38 is upregulated in PBMC of ZSDs and X-ALD patients. Representative immunoblots for p-p38 in control (CTL) and in ZSD and X-ALD patients. Protein levels are normalized respect to GAPDH. The histograms below show the p-p38 levels relative to CTL

In patients with ZSD, the expression of p-p38 was on overage 3.30-fold (range 1.56–4.26) higher than in controls. In particular, it was about fourfold higher in patients 1 and 3, respectively, and only 1.56 times in patient 2 who was in chronic therapy with vitamin E, coenzyme Q10, DHA. In patients with X-ALD the expression of p-p38 was on average 2 times greater than controls (range 1.21–2.84) (Fig. 1). No differences were observed according to the phenotypic subtype and treatment (transplant vs. no-transplant).

**Fig. 2:**
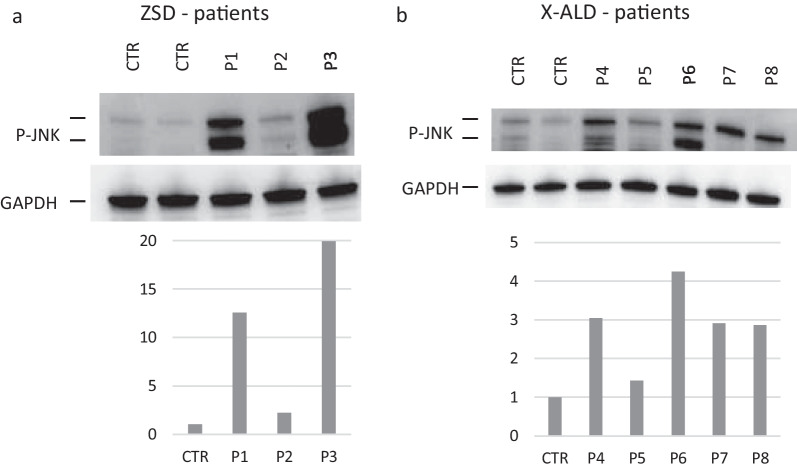
p-JNK is upregulated in PBMC of ZSDs and X-ALD patients. Representative immunoblots for p-JNK in control (CTL) and in ZSD and X-ALD patients. Protein levels are normalized respect to GAPDH. The histograms below show the p-JNK levels relative to CTL

The expression of p-JNK was on average 12-fold higher in ZSD patients (range 2.20–19.92) than in controls. It was 12 and 20 times greater in patients 1 and 3 respectively, and only 2 times greater in patient 2, who is on chronic therapy with vitamin E, coenzyme Q10, DHA. In patients with X-ALD it was on average 2.90- fold (range 1.43–4.24) greater than controls (Fig. 2). Again, no differences were noticed based on the phenotypic subtype or the treatment (transplant vs. no-transplant). There was a significant correlation between p-p38 and p-JNK values (r = 0.86).

### Autophagy

To test cells for autophagic activity, LC3 immunoblot analysis is most widely used. Endogenous LC3 is detected as two bands: one represents LC3-I, which is cytosolic (16 kDa), and the other LC3-II, which is conjugated with phosphatidylethanolamine and is present on isolation membranes and autophagosomes (14 kDa). The amount of LC3-II has been found to correlate with the number of autophagosomes, thus representing a good indicator of autophagosome formation [24].

**Fig. 3:**
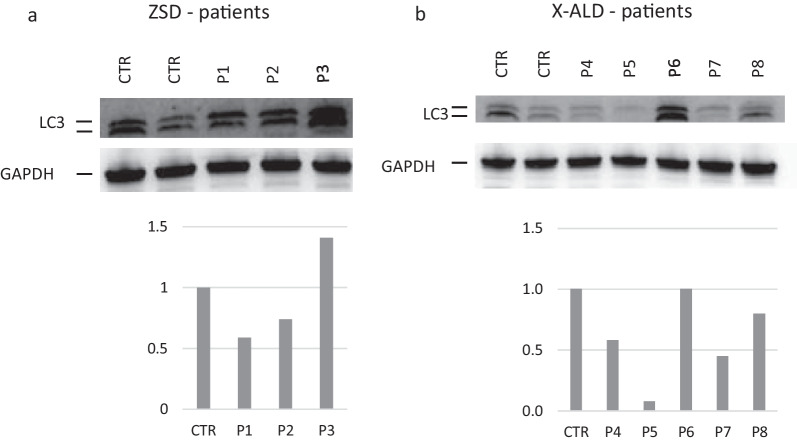
LC3. Autophagy is downregulated in PBMC of ZSDs and X-ALD patients. Representative immunoblots for LC3-I and LC3-II in control (CTL) and in X-ALD and ZSDs patients. Protein levels are normalized respect to GAPDH. The histograms below show the LC3-II/LC3-I ratio levels relative to CTL

We studied the autophagic flux by measuring LC3II/I ratio. In ZSDs patients, the values were reduced by 10% if compared with healthy controls without significant differences among the patients. A reduction by 40% was noticed in patients with X-ALD (Fig. 3). No significative differences were noticed based on phenotypic subtypes or treatment (transplant vs no transplant).

No correlation with the MAPK levels emerged.

## Discussion

Oxidative stress has been reported to have a significant role in the pathogenesis of peroxisomal disorders such as other neurodegenerative diseases. Lit is known on the intracellular activation of Mitogen-activated protein kinases (MAPKs) pathways in the peroxisomal disorders. Our results demonstrate that X-ALD and ZSD patients present an abnormal activation of MAPK pathway, showing higher values in ZSDs versus X-ALD patients.

MAPKs compose a family of protein kinases that play an essential role in relaying intracellular and extracellular signals, including ROS and inflammatory cytokines, to the nucleus via a cascade of phosphorylation events. There are three well-defined subgroups of MAPKs: the extracellular signal regulated kinases (ERKs), the c-Jun N-terminal kinases (JNKs), and the p38 MAPKs. Diverse cellular functions are regulated by MAPK signaling and the deleterious consequences of sustained activation of MAPK pathways may include uncontrolled proliferation, and unscheduled cell death. p38 and p-JNK activation has been implicated in the pathogenesis of several neurodegenerative diseases other than peroxisomal disorders [2, 25].

In addition to the inflammatory mediators, H_2_O_2_, produced by peroxisomes, activates MAPK pathways via activation of growth factor receptors and the oxidative modification of intracellular kinases. For example, thioredoxin becomes oxidized and disassociates from ASK-1 protein (MAP3K), leading to activation of JNK and p38 pathways. Moreover, intracellular H_2_O_2_ accumulation inactivates MAPKs phosphatases by oxidation of their catalytic cysteine, which leads to sustained activation of pathway [26].

Peroxisomes do not only generate ROS, but also interact with mitochondria, which may lead to further oxidative stress [23]. In particular, VLCFA accumulation may induce mitochondrial inner membrane depolarization and permeability transformation by replacing the lateral chains of phospholipid bilayers, which would disturb the proper functioning of oxidative phosphorylation system [2, 23, 27].

Finally, peroxisomal antioxidant defenses, enzymatic (catalase) and non-enzymatic (plasmalogen, DHA), can be compromised in peroxisomal disorders.

In patient P2, antioxidant treatment with vitamin E, DHA and Coenzyme Q10 appeared to reduce the activation of MAPK pathway. In fact, in the untreated ZSD patients (P1 and P3) the value of p-p38 was about 4 -fold higher than controls, while in P2 it was only 1.56. Accordingly, the value of p-JNK was about 12-fold higher in untreated ZSD patients than controls, but only twice as high in patient 2.

Our data showed that the induction of the p38 and JNK mediated MAPK pathways is attenuated by antioxidants, indicating the possibility for a new therapeutic option in ZSD patients and a possible use of MAPKs in PBMCs as biomarkers.

In our experience, the HSCT appears to have little effect on oxidative stress. Rockenbach et al. [16] demonstrated that parameters of oxidative stress in plasma of 4 X-ALD patients were reduced after HSCT, without achieving normalization. A limitation of our study is the small amount of time which has occurred since the transplant was performed in patient 7 and the lack of longitudinal data (pre-and post HSCT). Nevertheless, we can confirm the absence of a true normalization of MAPK activation after HSCT.

To maintain homeostasis in various internal and external stress responses and to remove oxidized products and damaged organelles, including oxidative stress, a correct autophagic machinery is essential [28]. Autophagy has been observed to be downregulated in X-ALD mouse model and human fibroblasts and could contribute to neurodegeneration [29, 30]. Our X-ALD patients showed a defective autophagy (LC3-II/LC3-I ratio), in accordance with what has been previously described. Moreover, we have demonstrated the same defect in our ZSD patients. Autophagic activity can be directly (e.g., through oxidative modification of autophagy-related proteins) or indirectly (e.g., through oxidative modification of transcription factors or signaling proteins, including MAPK) modulated by H_2_O_2_ [31]. Indeed, regarding PEX5-mediated pexophagy, H_2_O_2_ production can decrease the intracellular levels of the PEX5-ubiquitin, determining impairment of pexophagy itself [32]. Other factors besides oxidative stress can be involved in the blocking of autophagy. It has been demonstrated that VLCFAs accumulation destabilizes and increases the viscosity in model membranes [33]. This process can lead to a defect in the fusion of autophagosomes and lysosomes and impaired autophagy, as demonstrated in human fibroblasts [29, 34].

### Study limitations

Peroxisomal disorders are rare and clinically heterogeneous. Our clinical population is diverse in regard of its clinical phenotype and treatment received. This allows us to study the effect of therapies in single patients, but studies on larger populations are required in the future.

A limitation of our study is the lack of longitudinal data that may reflect the evolution of disease and the impact of the therapies. Longitudinal studies on early- treated patients will allow to better evaluate the correlation between said biomarkers, the clinical phenotype and the efficacy of the therapies.

## Conclusion

Our study demonstrates a greater activation of MAPK pathways and an inhibition of autophagy in patients with Zellweger spectrum disorders and X-linked adrenoleukodystrophy. Considering the absence of successful therapies and the growing interest towards new therapies with antioxidants and autophagy inducers, the identification and validation of biomarkers to monitor optimal dosing and biological efficacy of the treatments is of prime interest. Further studies on larger and earlier treated population are warranted.

## Data Availability

All data generated or analyzed during this study are included in this published article.

## Abbreviations

AMN: Adrenomyeloneuropathy
C26:0-lysoPC: C26:0-Lysophosphatidylcholine
CALD: Cerebral X-ALD
CNS: Central nervous system
DHA: Docosahexaenoic acid
GTO/GTE: Glycerol trioleate/glycerol trierucate
HSCT: Hematopoietic stem cell transplantation
IQ: Intelligence quotient
LC–MS/MS: Liquid chromatography tandem mass spectrometry
MAPK: Mitogen-activated protein kinases
MRI: Magnetic resonance imaging
ROS: Reactive oxygen species
PBD: Peroxisome biogenesis disorders
PBMC: Peripheral blood mononuclear cells
VLCFA: Very long chain fatty acid
X-ALD: X-linked adrenoleukodystrophy
ZSD: Zellweger spectrum disorders
